# Pallidal stimulation as treatment for camptocormia in Parkinson’s disease

**DOI:** 10.1038/s41531-020-00151-w

**Published:** 2021-01-18

**Authors:** Yijie Lai, Yunhai Song, Daoqing Su, Linbin Wang, Chencheng Zhang, Bomin Sun, Jorik Nonnekes, Bastiaan R. Bloem, Dianyou Li

**Affiliations:** 1 Department of Neurosurgery, Ruijin Hospital, Shanghai Jiao Tong University School of Medicine, Shanghai, China; 2 Neurosurgery Department, Shanghai Children’s Medical Center Affiliated to the Medical School of Shanghai Jiao Tong University, Shanghai, China; 3 Department of Neurosurgery, Liaocheng People’s Hospital and Liaocheng Clinical School of Shandong First Medical University, Liaocheng, China; 4 Department of Rehabilitation, Radboud University Medical Center, Donders Institute for Brain Cognition and Behavior, Nijmegen, The Netherlands; 5 Department of Neurology, Radboud University Medical Center, Donders Institute for Brain Cognition and Behavior, Nijmegen, The Netherlands

**Keywords:** Parkinson's disease, Neurological manifestations

## Abstract

Camptocormia is a common and often debilitating postural deformity in Parkinson’s disease (PD). Few treatments are currently effective. Deep brain stimulation (DBS) of the globus pallidus internus (GPi) shows potential in treating camptocormia, but evidence remains limited to case reports. We herein investigate the effect of GPi-DBS for treating camptocormia in a retrospective PD cohort. Thirty-six consecutive PD patients who underwent GPi-DBS were reviewed. The total and upper camptocormia angles (TCC and UCC angles) derived from video recordings of patients who received GPi-DBS were used to compare camptocormia alterations. Correlation analysis was performed to identify factors associated with the postoperative improvements. DBS lead placement and the impact of stimulation were analyzed using Lead-DBS software. Eleven patients manifested pre-surgical camptocormia: seven had lower camptocormia (TCC angles ≥ 30°; TCC-camptocormia), three had upper camptocormia (UCC angles ≥ 45°; UCC-camptocormia), and one had both. Mean follow-up time was 7.3 ± 3.3 months. GPi-DBS improved TCC-camptocormia by 40.4% (angles from 39.1° ± 10.1° to 23.3° ± 8.1°, *p* = 0.017) and UCC-camptocormia by 22.8% (angles from 50.5° ± 2.6° to 39.0° ± 6.7°, *p* = 0.012). Improvement in TCC angle was positively associated with pre-surgical TCC angles, levodopa responsiveness of the TCC angle, and structural connectivity from volume of tissue activated to somatosensory cortex. Greater improvement in UCC angles was seen in patients with larger pre-surgical UCC angles. Our study demonstrates potential effectiveness of GPi-DBS for treating camptocormia in PD patients. Future controlled studies with larger numbers of patients with PD-related camptocormia should extend our findings.

## Introduction

Camptocormia, an abnormal uncontrollable forward flexion of the spine while standing or walking, is a common type of postural deformity with an overall incidence of 5–19% in patients with Parkinson’s disease (PD)^1–3^. This deformity is often debilitating and can hinder patients during walking or performing activities of daily living^4^. Currently, few treatments are available for camptocormia in PD. Levodopa or botulinum toxin injection may be partially effective but the efficacy varies, and the majority of the patients are not helped satisfactorily by these approaches^1,2^. Dopamine agonists can even aggravate or induce camptocormia^2,5^.

In recent years, deep brain stimulation (DBS) has attracted increasing attention as a potential treatment of postural deformities in PD patients. Subthalamic nucleus (STN) and globus pallidus internus (GPi) are the two main targets of DBS for PD^6^. Various studies have reported the clinical effectiveness of STN-DBS in treating PD postural deformities. Recently, results from studies with large sample sizes showed that STN-DBS had a relatively small but significant therapeutic effect on abnormal posture^7,8^ and could especially bring about large improvement to those who were complicated with camptocormia^9^. Based on its efficacy in the treatment of primary dystonia, GPi-DBS has also been proposed to be effective for dystonic posture in PD^10^. However, compared to STN-DBS, the evidence for any efficacy of GPi-DBS for treating PD-related camptocormia has been limited to case reports with incongruent results^4,11–13^. Besides, the methods for measuring postural deformities varied between studies and the international consensus for determining patients’ flexion angle was not reached until recently^3,9,14^. Larger studies with consensus-based methods are therefore required to determine the effectiveness of GPi-DBS on camptocormia in PD patients.

Here we report the effectiveness of GPi-DBS on camptocormia in patients with PD based on a retrospective cohort of thirty-six consecutive subjects who underwent GPi-DBS. The effect of pre-surgical medication on total camptocormia or upper camptocormia (TCC/UCC) angles, as defined in the recent consensus for the measurement of the camptocormia angle^3^, was investigated by comparing the angles during the medication-OFF state (med-OFF) with angles during the medication-ON state (med-ON) before surgery. The benefit of DBS surgery was determined by comparing these angles during the pre-surgical med-OFF state with the angles during post-surgical med-OFF and stimulation-ON (med-OFF/DBS-ON) state. We also looked for factors that were potentially associated with post-surgical camptocormia angle improvements.

## Results

### Characteristics of included patients

Thirty-six consecutive patients were included in this retrospective study. The demographical data are presented in Table 1 and the results of motor assessment are presented in Table 2. Eleven (30.6%) of these 36 patients had camptocormia, in whom 7 (19.4%) patients were diagnosed with TCC-camptocormia (or lower camptocormia) as presenting with a TCC angle of ≥30° and 3 (8.3%) patients were diagnosed with UCC-camptocormia (or UCC) as presenting with a UCC angle of ≥45°. One (2.8%) patient presented with both TCC-camptocormia and UCC-camptocormia.

**Table 1:** Characteristics of included patients.

Characteristics	Value
*n*	36
Age at surgery (years)	63.7 ± 8.6
Gender	15 F/21 M
Age at PD onset	52.9 ± 9.2
Duration of PD (years)	10.8 ± 4.4
Follow-up time (months)	7.3 ± 3.3
LEDD (mg)	675.1 ± 275.3
Stimulation parameters	
Amplitudes (V)	L 3.1 ± 0.5/R 3.0 ± 0.6
Frequency (Hz)	L 134.1 ± 30.5/R 134.9 ± 30.6
Pulse width (μsec)	L 71.3 ± 13.0/R 72.3 ± 12.7

**Table 2:** Pre-surgical examinations at med-OFF state.

Items	Overall	Without CC	TCC-CC	UCC-CC
*n*	36	25	8	4
MDS-UPDRS III				
Total	54.0 ± 18.3	49.5 ± 16.0	69.9 ± 19.2	48.8 ± 11.7
Tremor	7.1 ± 5.9	6.6 ± 5.8	9.5 ± 5.5	5.8 ± 6.8
Rigidity	11.7 ± 5.3	11.1 ± 5.0	14.8 ± 5.8	8.0 ± 3.9
Bradykinesia	24.6 ± 8.6	22.5 ± 8.3	31.3 ± 7.1	24.0 ± 6.5
Axial	10.6 ± 4.7	9.2 ± 4.2	14.4 ± 5.4	11.0 ± 3.2
Posture	2.1 ± 1.3	1.6 ± 1.3	3.3 ± 0.7	2.8 ± 0.5
Camptocormia angles				
TCC angle	21.7 ± 11.6	15.9 ± 5.4	39.1 ± 10.1	25.4 ± 5.7
UCC angle	36.4 ± 7.1	34.2 ± 4.5	38.2 ± 9.2	50.5 ± 2.6
TCC-camptocormia	7	0	7	–
UCC-camptocormia	3	0	–	3
Both camptocormia^a^	1	0	1	1

*TCC* total camptocormia angle, *TCC-camptocormia* group of patients with a clinically diagnosed camptocormia as defined by a TCC angle ≥ 30°, *UCC* upper camptocormia angle, *UCC-camptocormia* group of patients with a clinically diagnosed camptocormia as defined by a UCC angle ≥ 45°.

^a^Both camptocormia means presenting with both TCC- and UCC-camptocormia.

### Effect of levodopa treatment on posture angles in the overall population and in patients with/without camptocormia

**Fig. 1:**
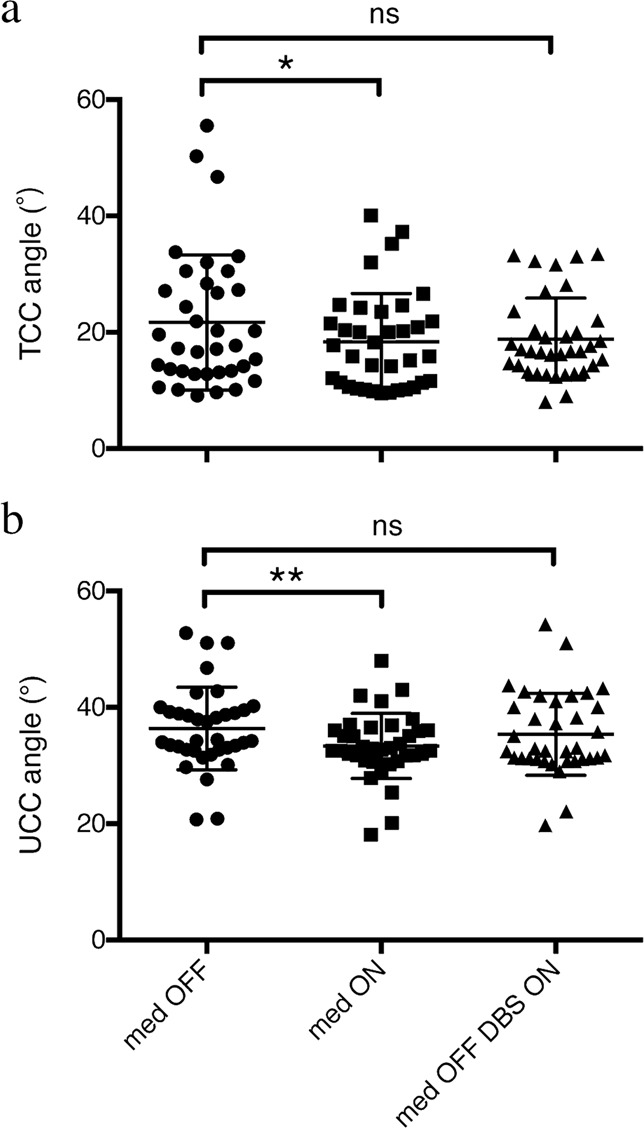
Effect of levodopa and surgery on camptocormia angles in the whole population. Pre- and post-surgical total- (**a**) and upper (**b**) camptocormia angles (TCC/UCC angles) were shown for individual PD patient. *p < 0.05; **p < 0.01; n.s.: not significant.

Pre-surgically, small but significant improvement was observed in the TCC angles (from 21.7° ± 11.6° to 18.4° ± 8.3°, *p* = 0.0185) and the UCC angles (from 36.4° ± 7.1° to 33.4° ± 5.6°, *p* = 0.0012) in response to levodopa treatment (Fig. 1). In patients with TCC-camptocormia, both the TCC angles (from 39.1° ± 10.1° to 27.8° ± 8.3°, *p* = 0.0566) and the UCC angles (from 38.2° ± 9.2° to 34.0° ± 7.1°, *p* = 0.0905) showed a nonsignificant reduction after administration of levodopa. In patients with UCC-camptocormia, a significant improvement was seen in the UCC angles (from 50.5° ± 2.6° to 36.3° ± 8.5°, *p* = 0.0317) but not with the TCC angles (from 25.4° ± 5.7° to 22.7° ± 9.1°, *p* = 0.4114). In patients without camptocormia, improvement in the TCC angles (from 15.9° ± 5.4° to 14.9° ± 5.1°, *p* = 0.0689) was not significant, whereas a small but significant reduction was seen in the UCC angles (from 34.2° ± 4.5° to 32.8° ± 4.5°, *p* = 0.0032).

### Effect of GPi-DBS on posture angles in the overall population and in patients with/without camptocormia

**Fig. 2:**
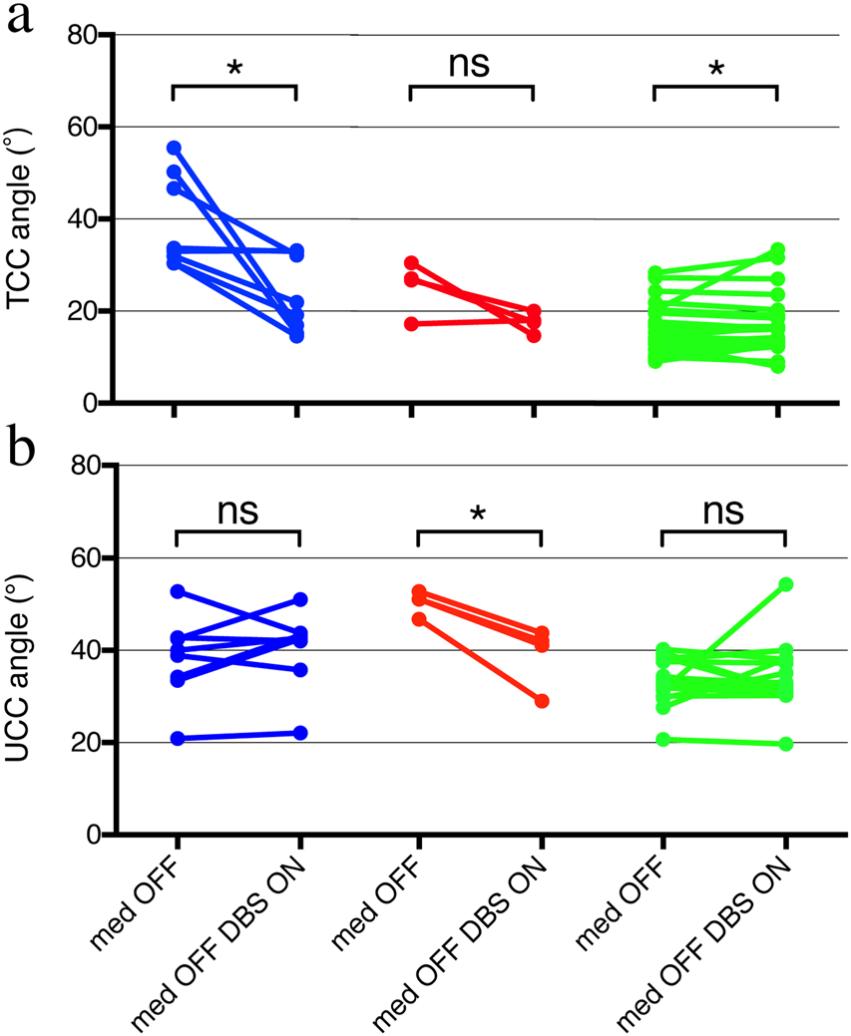
Improvements in camptocormia angles after surgery. Colored dots represent pre- and post-surgical total (**a**) and upper (**b**) camptocormia angles (TCC/UCC angles) in patients with lower camptocormia (blue symbols and lines), upper camptocormia (red symbols and lines) and without camptocormia (green symbols and lines). *p < 0.05; n.s.: not significant.

At a mean follow-up of 7.3 ± 3.3 months, both the TCC angles (from 21.7° ± 11.6° to 18.8° ± 7.1°; *p* = 0.0976; Fig. 1a) and the UCC angles (from 36.4° ± 7.1° to 35.4° ± 7.1° (*p* = 0.4198; Fig. 1b) showed a nonsignificant decrease in all patients treated with bilateral GPi-DBS. In patients with TCC-camptocormia, the TCC angles significantly decreased from 39.1° ± 10.1° to 23.3° ± 8.2° (*p* = 0.0168; Fig. 2a), whereas no significant improvement was seen in the UCC angles (from 38.2° ± 9.2° to 40.4° ± 8.5° *p* = 0.3715; Fig. 2b); in the UCC-camptocormia group, significant improvement was seen in the UCC angles (50.5° ± 2.6° to 39.0° ± 6.7°, *p* = 0.0124; Fig. 2b) but not in the TCC angles (from 25.4° ± 5.7° to 17.6° ± 2.2°, *p* = 0.1073; Fig. 2a); in patients without camptocormia, a slight but significant deterioration was seen in the TCC angles (from 15.9° ± 5.4° to 17.3° ± 6.6°, *p* = 0.0308; Fig. 2a), whereas a nonsignificant improvement was found in the UCC angles (from 34.2° ± 4.5° to 33.5° ± 5.9°, *p* = 0.6261; Fig. 2b). In addition, the levodopa equivalent daily dosage (LEDD) significantly decreased from 675.1 ± 275.3 mg to 534.0 ± 221.7 mg (*p* < 0.001) after surgery in the whole population.

### Factors associated with DBS effectiveness

**Fig. 3:**
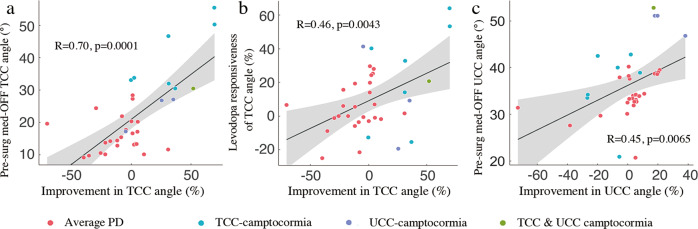
Relation between improvement in camptocormia angles and clinical variables. The improvement in TCC angles was significantly correlated with pre-surgical values of TCC angles (**a**) and its responsiveness to levodopa (**b**); improvement in UCC angles was found correlated with pre-surgical UCC angles. Gray areas represent the 95% CI. PD: Parkinson’s disease; TCC: total camptocormia; UCC: upper camptocormia; pre-surg: pre-surgical.

In the univariate analysis of the whole population, greater improvement in the TCC angles was found in patients with larger pre-surgical TCC angles during the med-OFF state (*p* = 0.0001; Fig. 3a) and better levodopa responsiveness of the TCC angle (*p* = 0.0043; Fig. 3b); improvement of the UCC angles were positively correlated with pre-surgical UCC angles (*p* = 0.0065; Fig. 3c). No significant correlation was found between percent improvement of TCC/UCC angles after surgery and the rest of the variables, including age at surgery, duration of PD, length of follow-up, baseline Movement Disorder Society-Sponsored Revision of the Unified Parkinson’s Disease Motion Assessment Scale Part III (MDS-UPDRS-III) total scores, percent improvement in MDS-UPDRS-III total scores in response to levodopa and levodopa responsiveness of the UCC angle (all *p*s > 0.05). In addition, in patients presented with TCC-camptocormia, values of pre-surgical TCC/UCC angles or levodopa responsiveness of TCC/UCC angles were not significantly correlated with the post-surgical improvements in TCC/UCC angles (all *p*s > 0.05). In the multivariate analysis incorporating aforementioned variables, pre-surgical TCC angles (*β* = 0.61, *p* = 0.0020) were identified as the independent predictor of post-surgical improvement in the TCC angles, whereas none of the rest of the variables remained predictive of the TCC/UCC angle improvements (all *p*s > 0.05).

**Fig. 4:**
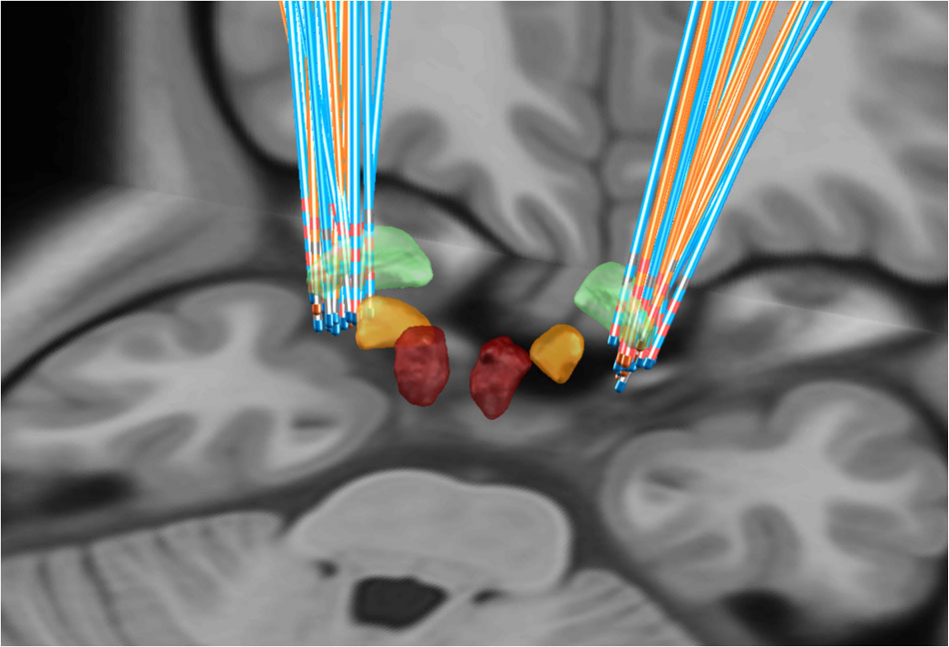
Reconstruction of the DBS electrodes. The electrodes of 10 patients with camptocormia (marked in orange) and 18 patients without camptorcormia (maked in blue) were shown on the T1-weighted Montreal Neurological Institute (MNI) template. Active contacts were marked in red. Masses with yellow described the location of the STN, red for the red nucleus, and green for the GPi.

**Fig. 5:**
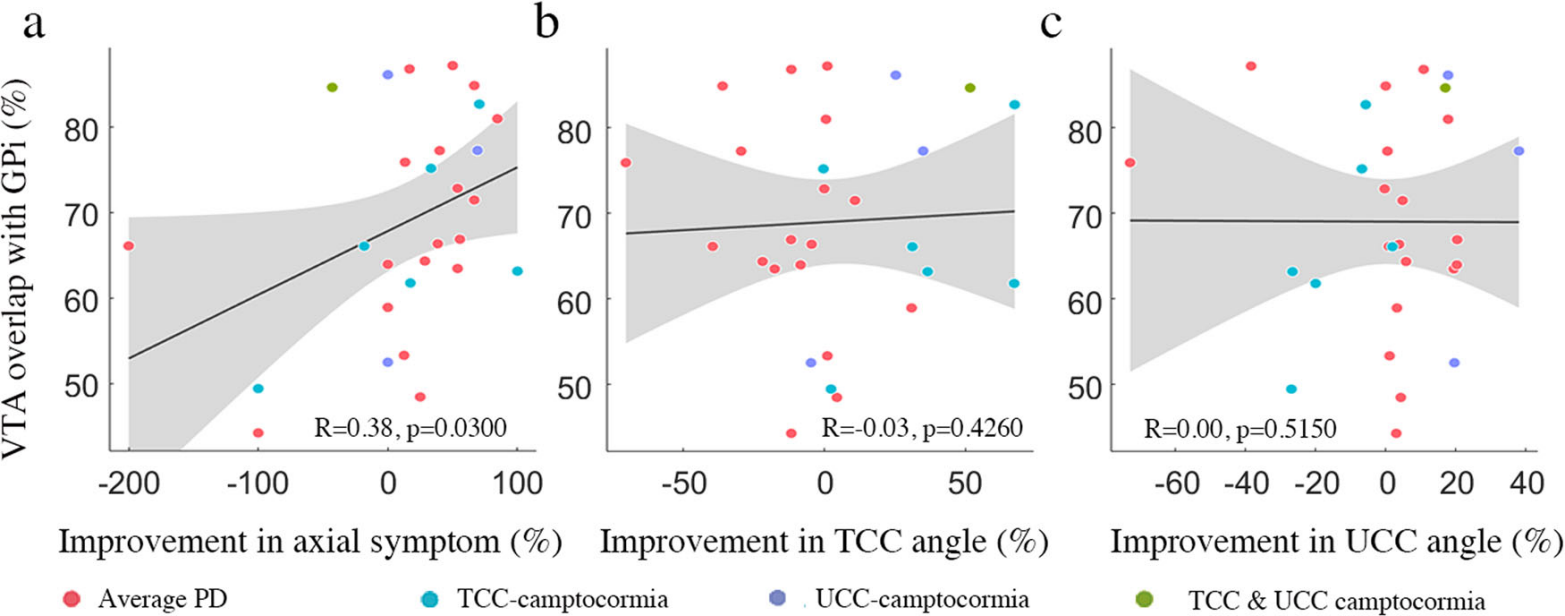
Correlation between percentage of VTA overlap with GPi and outcomes. The overlap volume significantly correlated with improvements in subscores for axial symptoms (**a**), but not with improvements in TCC angles (**b**) and UCC angles (**c**). Gray areas represent the 95% CI. PD: Parkinson’s disease; VTA: volume of tissue activated; TCC: total camptocormia; UCC: upper camptocormia.

**Fig. 6:**
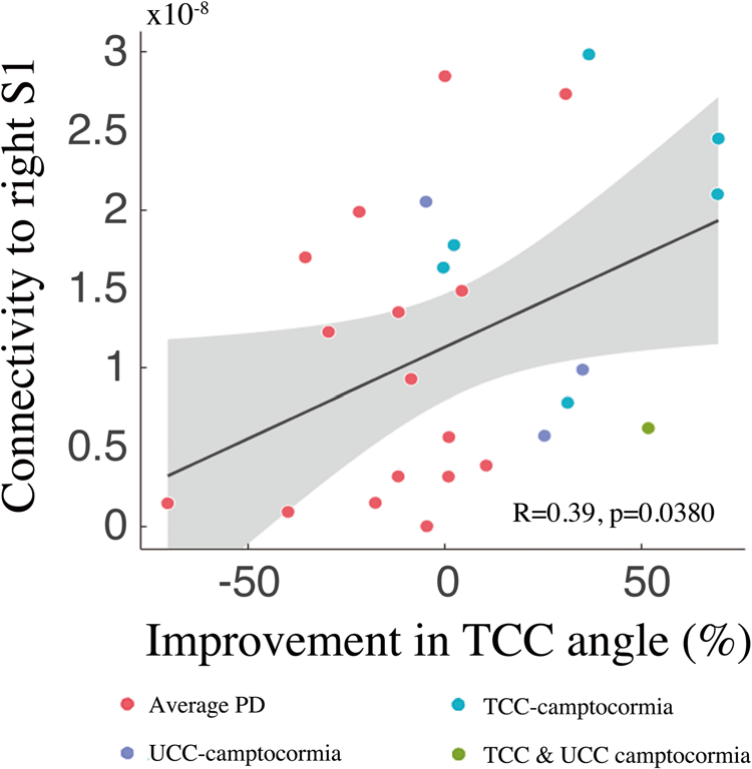
Significant correlation was found between structural connectivity from VTA to right S1 and percent improvement in TCC angles. Gray areas represent the 95% CI. PD: Parkinson’s disease; S1: somatosensory cortex; PD: Parkinson’s disease; TCC: total camptocormia; UCC: upper camptocormia.

To investigate the impact of different stimulation parameters on the outcome of GPi-DBS, a total of 28 patients (10 in 11 camptocormia patients) with available imaging and stimulation data were analyzed to reconstruct the location of DBS electrodes and model the stimulation impact. No significant outliers among the electrodes were identified through the manual examination (Fig. 4). In volume of tissue activated (VTA) analysis, although significant correlation was found between VTA overlap with GPi and percent improvements in axial subscores (*R* = 0.38, *p* = 0.0300; Fig. 5a), improvements in the TCC/UCC angles were not correlated with the volume of VTA intersection with GPi (all *p* > 0.05; Fig. 5b, c). By analyzing the possible fibers traversing through the VTA and projected to the volumetric space of the vast brain areas, we found the structural connectivity from VTA to right somatosensory cortex (S1) was significantly correlated with improvements in TCC angles (*R* = 0.39, *p* = 0.0380; Fig. 6).

## Discussion

This cohort study focused on the effectiveness of GPi-DBS for treating postural deformities in PD patients and its possible predictors. Pre-surgically, levodopa provided a small but significant improvement of bending angles in the whole population of PD patients, with an approximately equal effect on the measurement of TCC and UCC angles. The follow-up results showed that GPi-DBS can significantly improve postural alignments in PD patients with camptocormia, and the correlation analysis suggests that patients with larger pre-surgical TCC/UCC angles, better levodopa responsiveness of the TCC angle and higher connectivity from VTA to right S1 cortex could possibly gain greater benefits following surgery. These findings add to and extends previously published data in the aspect of clinical effectiveness and candidate selection of GPi-DBS for the treatment of camptocormia in PD^15,16^.

**Fig. 7:**
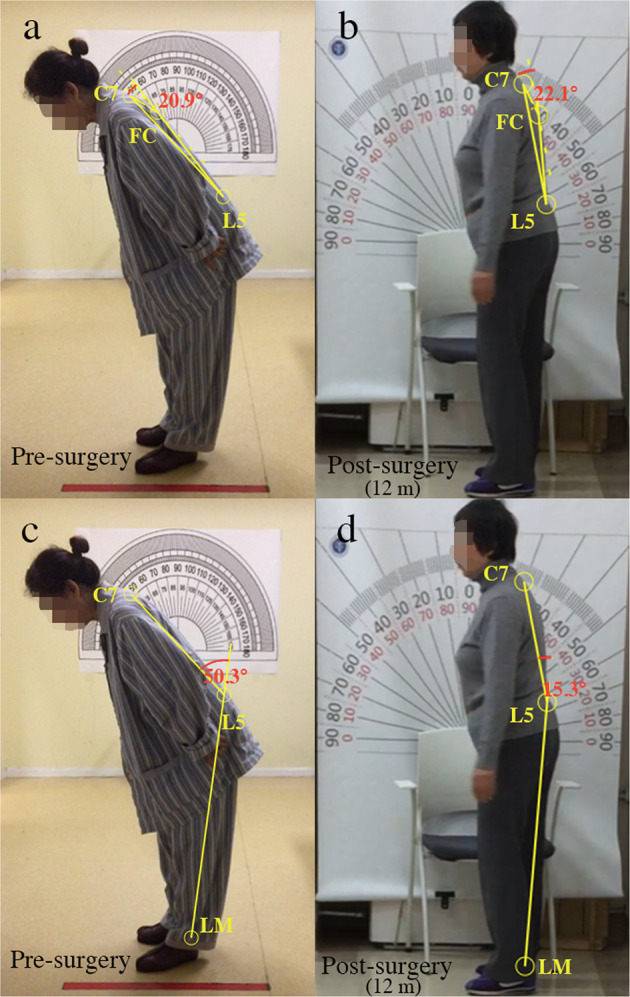
Measurement of the camptocormia angles angle in a patient with TCC-camptocormia pre- and post surgery. **a** Pre-surgical UCC angle; **b** UCC angle at 12 month post-surgery; **c** post-surgical TCC angle; **d** TCC angle at 12 months post-surgery. Written consent was obtained for publication of the photographs. C7: spinous process of vertebra C7; L5: suspected location of the spinous process of vertebra L5; LM: lateral malleolus; FC: vertebral fulcrum, the point with the greatest distance from the spine.

The effect of GPi-DBS on camptocormia could be described as a mean of 40.4% improvement seen for TCC-camptocormia and 22.8% for UCC-camptocormia; this marked improvement (around 16° in TCC and 11° in UCC on average) in patients with severe postural deformities showed important clinical utility (an example of patient with TCC-camptocormia before and after surgery can be seen in Fig. 7). This beneficial effect were comparable with previous reports on the treatment effect of GPi-DBS for PD-related camptocormia: in 2005, Micheli et al.^11^ reported a sustained improvement in camptocormia 6 months after GPi-DBS in a 62-year-old man with early PD symptoms; a 33% reduction in the Burke–Fahn–Marsden motor trunk subscore was observed 36 months after surgery in a patient with PD-related camptocormia^17^; Thani et al.^13^ utilized high-frequency neuromodulation of the GPi to successfully achieve relief of camptocormia in a 57-year-old woman with PD. However, despite a number of successful cases, treatment failure was also reported: in a patient whose camptocormia only minimally responded to dopaminergic medications, the immediate post-surgical alleviation after bilateral GPi-DBS did not sustained at the longer follow-up (15 months)^4^. In our study, improvements in the TCC/UCC angles ranged from −0.3% to 69.6% (mean 33.4%) in the subgroup of 11 patients with camptocormia, and similarly, a wide range of post-surgical improvements was also seen in the whole population. These findings suggest that the therapeutic effect of DBS on postural abnormalities could be promising in general, but the outcome may differ from person to person.

To date, much of the knowledge about the factors influencing the effectiveness of DBS on camptocormia were from studies on STN-DBS^7,9,18^. In 2018, a meta-analysis pooled the efficacy measures of five bilateral STN-DBS studies with an overall decrease in the mean sagittal plane bending angles from 56.6° ± 5.1° to 38.4° ± 6.6° after surgery and proposed duration of camptocormia of 2 years or less as predictive of better outcomes^9^. However, due to the retrospective nature of our study, we were not able to include duration of camptocormia as a covariate because the accurate time of camptocormia onset could not be retrieved^19^. Instead, we investigated the impact of PD disease duration on surgery outcome and found it was not correlated with the treatment effect of GPi-DBS. Preoperative levodopa responsiveness was another important factor suggested to be predictive of DBS effect on camptocormia^7,18^, although, as discussed in previous literature, the correlation between preoperative levodopa responsiveness and benefit from STN-DBS may be the result of methods used in statistical analysis and was not always congruent between studies^19–22^. In our univariate analysis of factors associated with improvements in camptocormia angles, the results showed that levodopa responsiveness of the TCC angle was positively correlated to DBS benefit. This suggests that the occurrence of camptocormia could be a type of off-period dystonia, in which favorable outcomes after DBS can be expected^18^. However, the above association found in our study still needs further validation as it did not reach significance in the following multivariate analysis. In both the univariate and multivariate analysis, larger pre-surgical TCC angles were suggested to predict higher post-surgical improvements. Similar findings were also reported in STN-DBS, which showed that patients with camptocormia were more likely to have a substantial improvement after STN-DBS compared to those with normal camptocormia angles^8^. These findings suggest that patients with better responsiveness to levodopa can be expected to gain larger improvements after DBS and camptocormia should not be a contraindication for DBS, but it might even benefit from the surgery.

It is important to note that, unlike tremor and rigidity, which are supposed to improve within minutes to hours, axial symptoms, such as postural abnormalities, usually require days and even months to improve after electrodes were turned on^23^. As a consequence, repeat programming is expected in order to achieve optimal relief and the stimulation parameters could therefore be highly influential in improvements in postural alignments^23^. However, despite brief documentation of stimulation parameters in some of the studies, few researchers investigated the effect of different sets of stimulation parameters on camptocormia^7,18^. Through the stimulation analysis, we modeled the effect of various stimulation settings on local regions. When correlating stimulation volume on GPi to clinical outcomes, in contrast to the axial subscore improvements, the overlap volume of VTA with GPi was not found significantly associated with improvement in camptocormia angles, which resembles recent findings that suggest the volume of VTA-GPi overlap may not positively related to outcomes of pallidal stimulation in dystonic symptoms^24^. Aside from its local effects on stimulated brain targets, DBS is also proposed to exert its therapeutic effect by modulating remote structures and the distributed brain networks^25^. In connectivity analysis, we found that the structural connectivity from VTA to right somatosensory cortex was significantly correlated with improvements in TCC angles, suggesting the role of somatosensory cortex and proprioceptive integration in mediating the effect of DBS treatment for camptocormia. In previous studies, proprioceptive disintegration was found highly related to postural deformities in PD and in patients with camptocormia^26,27^. Although the pathophysiology of camptocormia remains unclear, our findings indirectly lend support to the theory that postural control could require a complex system involving the integration of vestibular, visual, and proprioceptive sensory information^26^.

At the time of the study, there was no strong evidence for the superiority of STN-DBS over the GPi-DBS, or vice versa^28^. In our study, camptocormia was not the primary indication for surgery. The main motivation of choosing GPi as the preferred target was based on the concern of a possible cognitive impact of STN-DBS and also, due to the relatively low doses of levodopa administration in our current cohort (mean LEDD of 675.1 mg, 31 patients <1000 mg), the benefit of reducing medications, which is a key advantage for STN-DBS, was not a top priority^28,29^. Additional considerations were the need for less intensive monitoring of medication and stimulation adjustments, as previously suggested for most patients received GPi-DBS^29,30^. Therefore, the selection of the GPi as target was largely a pragmatic one. According to previous systematic reviews, there was also not enough evidence to adequately compare STN and GPi as the target for parkinsonian camptocormia or any of the postural deformities, e.g., Pisa syndrome and anterocollis, particularly because of the relatively small sample sizes^9,15^. Although compared to STN-DBS, impressive outcome (improvement of 50–100%) was seen in patients with dystonic camptocormia after GPi-DBS, this finding may not be easily replicated in PD, as dystonic camptocormia patients were younger, had shorter disease duration, and longer camptocormia duration^15^. Studies utilizing randomized designs are now required to provide stronger evidence for optimal target selection^8^.

There are several limitations of our study. First, the length of follow-up in the overall population was restricted to within 12 months after surgery. Though this narrowed follow-up period was adopted to prevent substantial disease progression during the study, it may not allow full improvement in dystonic symptoms, which can sometimes take months after optimal settings are found^23,31^. Also, as there are studies indicating that DBS might lost its initial beneficial effect at long term^4,22^, longitudinal studies with repeated assessments at longer follow-ups, e.g., 5 years post surgery, were needed to assess the effect of DBS on camptocormia taking the fact of disease progression into consideration^32^. Second, due to the potential for overfitting of the data, the relatively small number of participants limited our power in making inference based on the multivariate analysis^33^. Also, the mere four patients presented with UCC-camptocormia makes it difficult to draw firm conclusion on the effect of GPi-DBS in patients with UCC-camptocormia. To deal with this issue, multicenter studies could be expected for not only enlarging the sample size but also contributing in validating the reproducibility of results across datasets. Third, our study was retrospective in nature. Prospective and controlled studies therefore remain useful in investigating other predictive factors, including the duration of camptocormia^19^ and features in the electromyogram recordings of flexor and extensor muscles of the trunk^34^.

Our study demonstrates effectiveness of GPi-DBS in improving camptocormia in PD patients. Specifically, patients present with larger pre-surgical TCC/UCC angles, better pre-surgical responsiveness of TCC angles to levodopa, and higher VTA to S1 structural connectivity may experience larger improvement in posture. These findings suggest that camptocormia should not be a contraindication for DBS, but it might even improve following GPi-DBS. Further randomized controlled studies with repeat measurement and multicenter data could help determine the long-term effects of DBS, identify predictors of outcome, and validate the reproducibility of results across datasets.

## Methods

This study has been carried out in accordance with The Code of Ethics of the World Medical Association (Declaration of Helsinki). The ethics committee of the Ruijin Hospital Shanghai Jiao Tong University School of Medicine approved this retrospective clinical research. Written informed consent was obtained from all patients. The authors affirm that human research participants provided informed consent, for publication of the images in Fig. 7.

### Study population

PD patients who were treated with bilateral GPi-DBS at Ruijin Hospital from January 2017 to January 2019 with video-taped pre- and post-surgical assessments were retrospectively analyzed. A lateral view was obtained from the video for each assessed condition.

### Inclusion and exclusion criteria

The inclusion criteria were as follows: (a) PD in Hoehn and Yahr stages 2–4, (b) 40–75 years old, (c) available video-taped motor examinations for pre-surgical (med-ON and med-OFF) and post-surgical (med-OFF/DBS-ON) conditions within 1-year follow-up after surgery, and (d) bilateral GPi- DBS treatment.

The exclusion criteria were as follows: (a) other neurological disease or injuries which could affect gait and posture, (b) history of lesions or DBS of other brain targets or spinal surgery, (c) severe orthopedic spine injuries or diseases (such as vertebral fracture, severe osteoporosis, Pott’s disease, etc.), and (d) postural abnormalities caused by trauma or disease after DBS.

### Surgical procedure

Preoperatively, the location of the GPi was determined using a stereotactic computed tomography (CT) scan [with the Leksell (Elekta, Inc.) head frame] coregistered to high-resolution 3.0 T T1- and T2-weighted magnetic resonance imaging (MRI) images with Leksell SurgiPlan (Elekta, Stockholm, Sweden). In general, the target was defined directly under the guidance of coregistered image and the location was about 2–4 mm anterior to the midpoint of the anterior commissure–posterior commissure line (AC–PC), 18–22 mm lateral to the AC–PC line, and 2–4 mm below the AC–PC line. The procedure was performed under general anesthesia. After confirmation of location of the electrodes (Model 3387, Medtronic, Inc., Minneapolis, MN, USA; or Model L302, PINS, Inc., Beijing, China) with intraoperative CT, the impulse generator was implanted. A CT or MRI scan was performed 1 week after the surgery to confirm the location of the electrodes.

### Symptom assessment and computational methods

Pre-surgical evaluation was conducted 1–2 days before surgery and post-surgical evaluation was conducted during the corresponding follow-ups. The length of follow-up was restricted to within 12 months after surgery (median: 6 months; range: 1–12 months), during which a marked disease progression was unlikely to happen.

#### Posture analysis

Based on the method recommended in the consensus statement by Margraf et al.^3^, postural angles were determined by two blinded physicians with an analysis of lateral view pictures of each patient standing still with the camera lens at approximately waist level; discrepancies were solved during a consensus meeting. The photographs were marked as follows^3^: C7 (spinous process of vertebra C7), L5 (suspected location of the spinous process of vertebra L5), LM (lateral malleolus), and FC (vertebral fulcrum, the point with the greatest distance from the spine).

According to the above points, the camptocormia angles were calculated as follows: (a) TCC angle = the angle between the line from the LM to L5 and the line between L5 and C7, (b) UCC angle = the angle between the line from L5 to FC and the line from FC to C7. An online tool was used to calculate the angles (https://www.neurologie.uni-kiel.de/de/axial-posturale-stoerungen/camptoapp)^3^. Using the cut-off for severity of postural angles in previous studies (TCC angle ≥ 30° for lower camptocormia, or TCC-camptocormia and UCC angle ≥ 45° for UCC or UCC-camptocormia)^3,14^, whether TCC-camptocormia or UCC-camptocormia was present was determined. In addition, a clinical posture score using the item “posture” in the MDS-UPDRS-III was obtained.

##### Motor examination

MDS-UPDRS-III was used to assess the patients’ motor symptoms. Preoperatively, the evaluation was carried out in med-OFF state (12 hours’ discontinuation of levodopa and 72 h of other anti-Parkinson medication) and med-ON state (1.5 times routine drug use and 45 min after administration); after surgery, the evaluation was carried out at med-OFF/DBS-ON state.

##### Collection of clinical information

Age at surgery, gender, duration of PD, medication, stimulation parameters, and other medical histories were collected.

##### Based on the postoperative CT or MRI images

Position of the electrodes in the nucleus was reconstructed using the lead-DBS toolbox (version 2.2.3) on Matlab according to the methods described by Horn et al.^35^. The VTA was estimated with Lead-DBS based on finite element models. Conductivity values for white matter were set to 0.14 S/mm and for gray matter to 0.33 S/mm. Thresholding of the potential gradient at 0.2 V/mm then determined activated tissue^36^. GPi were located on the DISTAL atlas, as this atlas was designed for surgical targets in basal ganglia and was proved to be of high accuracy of localization^37^. Overlaps between VTAs and the GPi were calculated for both hemispheres and summed up and normalized with the total volume of GPi. For structural connectivity, the normative group connectome from 32 subjects of the Human Connectome Project at Massachusetts General Hospital (https://ida.loni.usc.edu/login.jsp) was used. These data were acquired on a specially designed MRI scanner with more powerful gradients than available on conventional MRI scanners. The processing steps were described previously^36^. In each subject, 200,000 fibers were sampled and transformed into MNI space. Structural connectivity was calculated as fibers traversing through the VTA and projected to the volumetric space of the brain in 2 mm isotropic resolution, denoting the portion of fibers (connected to the VTA) that traversed through each voxel^35,36^. Parcellation of motor cortices was based on the Human Motor Area Template atlas, in which primary motor cortex (M1), somatosensory cortex (S1), supplementary motor area (SMA), pre-SMA, lateral premotor cortex along the dorsal and ventral plane (PMd and PMv) were defined^38^.

### Statistical analysis

Data were described using means and SDs for continuous variables and frequencies for categorical variables. A two-tailed paired *t*-test was used to analyze the changes in TCC and UCC angles before and after operation. Univariate and multivariate analysis were used to evaluate the association between clinical/demographic characteristics and the extent of the effect of GPi-DBS on camptocormia angles, which was measured using the percentage changes (pre-surgical med-OFF vs. post-surgical med-OFF/DBS-ON evaluation) of the TCC and UCC angles. Age at surgery, gender, duration of PD, follow-up time, pre-surgical motor score (MDS-UPDRS-III) at med-OFF, relative response to levodopa in motor score, and TCC/UCC angles (pre-surgical med-OFF vs. pre-surgical med-ON evaluation) were explored as covariates of interest. Two-sided *p*-values < 0.05 were considered significant. STATA 14.0 was used to analyze the data.

### Reporting summary

Further information on research design is available in the Nature Research Reporting Summary linked to this article.

## Supplementary information

Reporting Summary

## Data Availability

The data that support the findings of this study are available from the corresponding author upon reasonable request.
