# Author Correction: Pallidal stimulation as treatment for camptocormia in Parkinson’s disease

**DOI:** 10.1038/s41531-021-00178-7

**Published:** 2021-03-29

**Authors:** Yijie Lai, Yunhai Song, Daoqing Su, Linbin Wang, Chencheng Zhang, Bomin Sun, Jorik Nonnekes, Bastiaan R. Bloem, Dianyou Li

**Affiliations:** 1 Department of Neurosurgery, Ruijin Hospital, Shanghai Jiao Tong University School of Medicine, Shanghai, China; 2 Neurosurgery Department, Shanghai Children’s Medical Center Affiliated to the Medical School of Shanghai Jiao Tong University, Shanghai, China; 3 Department of Neurosurgery, Liaocheng People’s Hospital and Liaocheng Clinical School of Shandong First Medical University, Liaocheng, China; 4 Department of Rehabilitation, Radboud University Medical Center, Donders Institute for Brain Cognition and Behavior, Nijmegen, The Netherlands; 5 Department of Neurology, Radboud University Medical Center, Donders Institute for Brain Cognition and Behavior, Nijmegen, The Netherlands

**Keywords:** Parkinson's disease, Neurological manifestations

Correction to: *npj Parkinson’s Disease* 10.1038/s41531-020-00151-w.

The original version of this Article contained an error in the author affiliations.

Affiliation number 1 incorrectly read “Center for Functional Neurosurgery, Department of Neurosurgery, Ruijin Hospital, Shanghai Jiao Tong University School of Medicine, Shanghai, China”.

This has now been corrected in both the PDF and HTML versions of the Article.

