# Supplementary material for: Pallidal stimulation as treatment for camptocormia in Parkinson’s disease
Source: NPJ Parkinsons Dis. 2021 Jan 18;7:8. doi: 10.1038/s41531-020-00151-w (PMC7813849; doi:10.1038/s41531-020-00151-w)
Supplement: Supplementary file 1 — Reporting Summary [file 41531_2020_151_MOESM1_ESM.pdf]

nature research

Corresponding author(s): Dianyou Li

Last updated by author(s): Aug 18, 2020

Reporting Summary

Nature Research wishes to improve the reproducibility of the work that we publish. This form provides structure for consistency and transparency in reporting. For further information on Nature Research policies, see our Editorial Policies and the Editorial Policy Checklist.

Statistics

For all statistical analyses, confirm that the following items are present in the figure legend, table legend, main text, or Methods section.

n/a	Confirmed	
☐	☒	The exact sample size (n) for each experimental group/condition, given as a discrete number and unit of measurement
☐	☒	A statement on whether measurements were taken from distinct samples or whether the same sample was measured repeatedly
☐	☒	The statistical test(s) used AND whether they are one- or two-sided<br>*Only common tests should be described solely by name; describe more complex techniques in the Methods section.*
☐	☒	A description of all covariates tested
☐	☒	A description of any assumptions or corrections, such as tests of normality and adjustment for multiple comparisons
☐	☒	A full description of the statistical parameters including central tendency (e.g. means) or other basic estimates (e.g. regression coefficient) AND variation (e.g. standard deviation) or associated estimates of uncertainty (e.g. confidence intervals)
☐	☒	For null hypothesis testing, the test statistic (e.g. F, t, r) with confidence intervals, effect sizes, degrees of freedom and P value noted<br>*Give P values as exact values whenever suitable.*
☒	☐	For Bayesian analysis, information on the choice of priors and Markov chain Monte Carlo settings
☒	☐	For hierarchical and complex designs, identification of the appropriate level for tests and full reporting of outcomes
☒	☐	Estimates of effect sizes (e.g. Cohen's d, Pearson's r), indicating how they were calculated

Our web collection on statistics for biologists contains articles on many of the points above.

Software and code

Policy information about availability of computer code

Data collection	no software was used
Data analysis	STATA 14.0, LeadDBS v2.2.3

For manuscripts utilizing custom algorithms or software that are central to the research but not yet described in published literature, software must be made available to editors and reviewers. We strongly encourage code deposition in a community repository (e.g. GitHub). See the Nature Research guidelines for submitting code & software for further information.

Data

Policy information about availability of data

All manuscripts must include a data availability statement. This statement should provide the following information, where applicable:

- Accession codes, unique identifiers, or web links for publicly available datasets
- A list of figures that have associated raw data
- A description of any restrictions on data availability

The data that support the findings of this study are available from the corresponding author upon reasonable request.

Field-specific reporting

Please select the one below that is the best fit for your research. If you are not sure, read the appropriate sections before making your selection.

☒ Life sciences ☐ Behavioural & social sciences ☐ Ecological, evolutionary & environmental sciences

For a reference copy of the document with all sections, see nature.com/documents/nr-reporting-summary-flat.pdf

Life sciences study design

All studies must disclose on these points even when the disclosure is negative.

Sample size	Data was retrospective collected from Ruijin Hospital and the sample size was the number of patients who met the inclusion criteria of the study.
Data exclusions	Patients without available imaging and stimulation parameters at follow-up were excluded in the imaging analysis. No patient data was excluded from the effectiveness study.
Replication	We collected all participants information, including age at surgery, gender, duration of PD, follow-up time, per-surgical motor score (MDS-UPDRS-III) at med-OFF, relative response to levodopa in motor score and TCC/UCC angles (per-surgical med-OFF vs. per-surgical med-ON evaluation). And further used them as covariates during the analyses.
Randomization	This is a retrospective single-armed observational study where randomization was not applicable.
Blinding	Postural angles were determined by two blinded physicians with an analysis of lateral view pictures of each patient standing still with the camera lens at approximately waist level; discrepancies were solved during a consensus meeting.

Reporting for specific materials, systems and methods

We require information from authors about some types of materials, experimental systems and methods used in many studies. Here, indicate whether each material, system or method listed is relevant to your study. If you are not sure if a list item applies to your research, read the appropriate section before selecting a response.

Materials & experimental systems

n/a	Involved in the study	
☒	☐	Antibodies
☒	☐	Eukaryotic cell lines
☒	☐	Palaeontology and archaeology
☒	☐	Animals and other organisms
☐	☒	Human research participants
☒	☐	Clinical data
☒	☐	Dual use research of concern

Methods

n/a	Involved in the study	
☒	☐	ChIP-seq
☒	☐	Flow cytometry
☐	☒	MRI-based neuroimaging

Human research participants

Policy information about studies involving human research participants

Population characteristics	Thirty-six patients were included in this retrospective analysis, the mean age at surgery was 63.7±8.6 years, 15 females, the mean age at Parkinson's disease (PD) onset was 52.9±9.2 years, duration of PD was 10.8±4.4 years and the mean follow-up time was 7.3±3.3 months post-surgery.
Recruitment	Participants were recruited based on their clinical visit for surgery.
Ethics oversight	The ethics committee of the Ruijin Hospital Shanghai Jiao Tong University School of Medicine approved this retrospective clinical research.

Note that full information on the approval of the study protocol must also be provided in the manuscript.

Magnetic resonance imaging

Experimental design

Design type	Preoperative imaging

Design specifications	Single block with T1 and T2 structural imaging.
Behavioral performance measures	Not applicable.

Acquisition

Imaging type(s)	Structural
Field strength	3.0T
Sequence & imaging parameters	Structural images were acquired using a T1WI magnetization-prepared rapid gradient-echo sequence (MPRAGE) with the following parameters: repetition time (TR)=6.5 s, echo time (TE)=2.0 ms, slice thickness=2.0 mm, flip angle=15°, field of view (FOV)=240 mm x240 mm; and also a T2WI fast spin echo sequence (FSE) with the following parameters: TE=127.5 ms, TR=3 000.0 ms, slice thickness=2.0 mm, flip angle=90°, FOV=240 mm x240 mm.
Area of acquisition	Whole brain

Diffusion MRI ☐ Used ☒ Not used

Preprocessing

Preprocessing software	Lead-DBS v2.2.3
Normalization	Patient-to-template normalization were using non-linear advanced normalization tools (ANTs) implemented in LeadDBS, and within patient (e.g. T2 to T1) normalization was using linear method in ANTs.
Normalization template	MNI 152
Noise and artifact removal	According to Lead-DBS, when a patient folder is loaded in Lead-DBS, a bias-field correction step based on the N4 algorithm is automatically applied to all preoperative MRI sequences.
Volume censoring	The raw images and co-registration were checked manually to ensure quality and accuracy.

Statistical modeling & inference

Model type and settings	Not applicable. In this study, the pre-surgical MRI imaging were only used for localization of the electrode but not for statistical comparisons.
Effect(s) tested	Not applicable.

Specify type of analysis: ☐ Whole brain ☒ ROI-based ☐ Both

Anatomical location(s)	GPi were located on the DISTAL atlas, as this atlas was designed for surgical targets in basal ganglia and was proved to be of high accuracy of localization. Pacellation of motor cortices was based on the Human Motor Area Template (HMAT) atlas, in which primary motor cortex (M1), somatosensory cortex (S1), supplementary motor area (SMA), pre-SMA, lateral premotor cortex along the dorsal and ventral plane (PMd and PMv) were defined.
Statistic type for inference (See Eklund et al. 2016)	Not applicable.
Correction	Not applicable.

Models & analysis

n/a	Involved in the study
☒	☐ Functional and/or effective connectivity
☒	☐ Graph analysis
☒	☐ Multivariate modeling or predictive analysis
